# Clinical assessment of upper limb impairments and functional capacity in Parkinson's disease

**DOI:** 10.1055/s-0044-1786763

**Published:** 2024-05-13

**Authors:** Naina Joshi, Komalpreet Saini, Lobsang Chonzom

**Affiliations:** 1 Lovely Professional University, Department of Physiotherapy and Paramedical Sciences, Punjab, India.

Dear editor,

First and foremost, we express our gratitude towards the authors for their clear and concise description of the article titled “Clinical Assessment of upper limb impairments and functional capacity in Parkinson's Disease: a systematic review”. 1 While the study addresses an important gap in Parkinson disease (PD) research, particularly in the rehabilitation field, certain limitations and areas of improvement need to be addressed for the research to have a more substantial impact.

To begin with, there is a lack of clarity in the objective. The article's objective is to “access functional capacity.” While this objective is essential, the article does not clearly define what is meant by “functional capacity” in the context of upper limb assessments. According to our view, “functional capacity” refers to a person's ability to carry out tasks and activities that are desired or required in their lives, and under-regulated settings. These activities differ from person to person. A clear and concise definition of this term would help readers better understand the scope and goals of the study. 1 There is a lack of clarity in the objective because, in the abstract, the main goal was to determine the precise outcome to measure upper limb function in PD and to access functional capacity. However, another goal was added later to the main article, which is currently undergoing revision, and the distinction between primary and secondary outcomes was not made.

The only database searched was PubMed; therefore, the result of the study cannot be deemed true. According to the Preferred Reporting Items for Systematic Reviews and Meta-Analyses (PRISMA) checklist, different research designs, such as observational and experimental studies, and quantitative studies, such as clinical trials, meta-analyses, systematic reviews, and case reports were mentioned. 2 However, PubMed was mentioned as the only search engine under the heading “search strategy and selection criteria,” while Figure 1 showed 12 additional records that were found in other sources as well, but the names of such sources were not mentioned.

Moreover, there was incomplete acknowledgment of language. The article mentions that it only considered literature published in English, which could introduce language bias.

Apart from this, the abstract and the results section mention 2.239 participants, which certainly caused confusion among readers, as the number of participants can never be expressed in decimals. 3 Instead of using a decimal period, a decimal comma should have been used for the number of participants: “2,239”.

Another point that needs to be noted is that neither the study risk of bias assessment nor effect measures were mentioned, which is essential for the synthesis or presentation of results.

There was also a limited discussion of the clinical implications. The conclusion suggests that there is a shortage of specific tests to assess the functional capacity of the upper limbs in PD. While this is an important observation, the article does not delve into the potential clinical implications of this shortage. How does this lack of specific outcome measures impact the treatment and rehabilitation of PD patients? This aspect should be explored in greater detail.

moreover, there was a lack of forward-looking recommendations. The article concludes by highlighting the insufficiency in assessing the functional capacity of the upper limbs. However, it would be beneficial to provide recommendations or insights into how this issue can be addressed in future research or the clinical practice. Offering practical suggestions to develop specific outcome measures would enhance the article's usefulness to the rehabilitation community. The authors should acknowledge this limitation and discuss potential implications for the comprehensiveness of their findings.
